# Pig nasal and rectal microbiotas are involved in the antibody response to *Glaesserella parasuis*

**DOI:** 10.1038/s41598-025-85867-6

**Published:** 2025-01-17

**Authors:** Pau Obregon-Gutierrez, Yasser Mahmmod, Emili Barba-Vidal, Marina Sibila, Florencia Correa-Fiz, Virginia Aragón

**Affiliations:** 1 Unitat Mixta d’Investigació IRTA-UAB en Sanitat Animal, Centre de Recerca en Sanitat Animal (CReSA), Campus de la Universitat Autònoma de Barcelona (UAB), 08193 Bellaterra, Barcelona, Spain; 2 Institut de Recerca I Tecnologia Agroalimentàries, Programa de Sanitat Animal, Centre de Recerca en Sanitat Animal (CReSA), Campus de la Universitat Autònoma de Barcelona (UAB), 08193 Bellaterra, Barcelona, Spain; 3 WOAH Collaborating Centre for the Research and Control of Emerging and Re-Emerging Swine Diseases in Europe (IRTA-CReSA), 08193 Bellaterra, Barcelona, Spain; 4 Department of Veterinary Clinical Sciences, College of Veterinary, Medicine, Long Island University, 720 Northern Boulevard, Brookville, NY 11548 USA; 5 HIPRA, Avda. La Selva, 135, 17170 Amer, Girona, Spain

**Keywords:** Pig, Swine, Microbiota, Vaccination, Antibody response, Computational biology and bioinformatics, Microbiology, Microbial communities, Vaccines

## Abstract

Vaccination stands as one of the most sustainable and promising strategies to control infectious diseases in animal production. Nevertheless, the causes for antibody response variation among individuals are poorly understood. The animal microbiota has been shown to be involved in the correct development and function of the host immunity, including the antibody response. Here, we studied the nasal and rectal microbiota composition in association with the antibody response against the pathobiont *Glaesserella parasuis*. The nasal and rectal microbiotas of 24 piglets were sampled in two farms before vaccination and in one unvaccinated farm (naturally exposed to the pathobiont) at similar time. Microbiota composition was inferred by V3V4 16S rRNA gene sequencing and bioinformatics analysis, and the antibody response was quantified using the variation between the levels before and after vaccination (normalized per farm). Piglets with higher antibody responses showed more diverse nasal and rectal microbial communities compared to piglets with lower responses. Moreover, swine nasal core microbiota colonizers were associated with higher antibody levels, such as several members from *Bacteroidales* and *Clostridiales* orders and genera including *Moraxella*, *Staphylococcus*, *Fusobacterium* and *Neisseria*. Regarding taxa found in the rectal microbiota, associations with antibody responses were detected only at order level, pointing towards a positive role for *Clostridiales* while negative for *Enterobacteriales*. Altogether, these results suggest that the microbiota is associated with the antibody response to *G. parasuis* (and probably to other pathogens) and serves as starting point to understand the factors that contribute to immunization in pigs.

## Introduction

Animals live in constant contact with a variety of pathogens, which are controlled by the immune system. Antibodies, which target specific pathogens, constitute one of the main effector responses of adaptative immunity^1^. The adaptive immune response can hold memory through antigen-specific memory cells, permitting a more effective response in subsequent encounters with the pathogen^1,2^. One of the best ways to stimulate immunization and memory against pathogens is vaccination^2^, which represents an efficacious strategy to control infectious diseases nowadays^3^. Vaccines have the potential to reduce disease severity, eliminate pathogens locally and even eradicate them globally^3^. In consequence, they also contribute to the reduction in the use of antibiotics, which is particularly needed for minimizing the emergence of multidrug resistant bacteria^4^. However, not all individuals exhibit the same level of response to vaccination. Several factors such as maternal immunity, host genetics and environmental factors, among others, can be responsible for vaccination failure^5^. Therefore, it is crucial to further explore why the immune response (either after natural exposure to the pathogen or after vaccination) is variable among individuals^4–8^, and to identify the factors that contribute to a robust response^7,8^.

The animal microbiota, the community of microorganisms inhabiting different niches of the animal host, is known to have an important role in the immune system maturation and modulation^7^. The microbiota is involved in the local immune response, such as the stimulation of immune cells with bacterial compounds in the intestine or airway mucosae, but also in systemic immune responses via dissemination of microbial products and/or immune signals and cells through the whole organism^7^. Moreover, the stated systemic effect can be enhanced by the constant crosstalk of the different microbiotas in an organism^9^. In agreement, poor or deficient immune responses in the context of microbiota dysbiosis have been reported^7^. The relationship between the microbiota and the immune response is still to be unveiled, making the study of the microbiota a key point in vaccination efficacy studies^10^.

Mounting evidence shows that microbiota composition influences responses to vaccination in humans^7,10–19^, where several studies showed that patients with disrupted microbiota tend to show a reduced response to vaccination^19,20^. In the case of swine, vaccination programs are essential to face the strong institutional call towards the reduction in the use of antibiotics in animal farming^21^ and are critical to control infectious diseases, such as Glässer’s disease^22^, an endemic disease caused by *Glaesserella parasuis*, a pathobiont member of the porcine nasal microbiota that colonizes young piglets early after birth^23^. Few studies assess the microbiota in relation with vaccine response in pigs. In 2019 and 2020, Munyaka et al. studied the fecal microbiota as a predictor of high and low vaccine response, measured by the levels of specific antibodies against *Mycoplasma hyopneumoniae* in serum^24,25^. They were able to identify several taxa that discriminated the high vaccine-responders and whose presence was positively correlated with antibody titters. In a different study, Sanglard, et al. showed that the composition of the vaginal microbiota of sows discriminated between high and low antibody-responders against a porcine reproductive and respiratory syndrome virus (PRRSV) vaccine^26^.

Here, using Glässer’s disease as a model where antibodies are important for protection, we studied the composition of the nasal and rectal microbiota in pigs and identified taxa associated with different level of antibodies after bacterin vaccination and/or natural exposure with the pathogen.

## Methods

### Samples included in the study

Twenty-four piglets were randomly selected from different litters from three Spanish farms (eight per farm). Two of the farms (farms 1 and 2) performed vaccination against Glässer’s disease with a commercial bacterin (HIPRASUIS® GLÄSSER) by injecting 2 ml/piglet at 3 and 6 weeks or at 2 and 5 weeks of age, respectively. In addition, in farm 2, sows were also vaccinated with the same vaccine before farrowing and penicillin was administered to the piglets at first day of life. To measure antibody levels, serum samples were taken within the 24 h before the first vaccination and 3 weeks after the second vaccination (9 and 8 weeks of age for farm 1 and 2, respectively). Samples were transported under refrigeration to the laboratory and were processed within 48 h after collection. Nasal and rectal microbiota samples were obtained from both nostrils and rectum using thin aluminum cotton swabs (Deltalab) taken before the first vaccination (together with the pre-vaccination serum samples). Similar times were used for the sampling of non-vaccinated pigs in a third farm (farm 3); i.e. microbiota samples at 1 week and serum samples at 1 and 9 weeks of age. Swabs were stored in 1000 µL DNA/RNA shield (Zymo Research) at 4ºC before further processing. These samples were also used in a previous study assessing gut-associated components of the nasal microbiota^27^.

### Antibody levels

Antibodies against *G. parasuis* were measured in serum using an in-house ELISA previously described^28^. Plates were coated overnight at 4ºC with 250 ng of F4 (a protein fragment from the outer membrane proteins VtaA of *G. parasuis*) in 50 µl of carbonate-bicarbonate buffer per well. After washing, wells were blocked with 1% casein in phosphate-buffered saline (PBS) with 0.05% Tween 20 (PBS-Tw20). Sera were diluted 1:100 in blocking solution and added to the wells. After 1 h of incubation at 37ºC, wells were washed and incubated with a goat anti-porcine IgG HRP-conjugated antibody (Sigma-Aldrich, Madrid, Spain) diluted 1:10,000. Positive reactions in the ELISA were developed using the 3,3,3,5-tetramethylbenzidine (TMB) substrate (Sigma-Aldrich, Madrid, Spain) and the reactions were stopped with 1 N sulfuric acid. Plates were then read in a Power Wave XS spectrophotometer (Biotech, Winooski, VT, USA) at 450 nm.

A preliminary classification into good (higher response) or bad (lower response) responders was performed in each group according to the variation between the initial level of antibodies and the level of antibodies at 8–9 weeks of age (delta antibody value, ΔAb). The piglets showing variations above the median of the group were considered as good responders and those below the median as bad responders. To deal with farm variability, this classification was done independently within each farm. However, among the 24 piglets included in the study, two piglets were not classified following these criteria. One piglet from farm 2 was considered a bad responder despite showing a ΔAb above the median, since the clear reduction showed in the level of antibodies (from 1.197 to 0.605; ΔAb =  − 0.592). For one piglet from farm 3, we did not have the initial levels of antibodies and therefore it was not included in the analyses using the ΔAb. Nevertheless, it was considered a bad responder according to its final level of antibodies in comparison to the rest of the animals in the same farm. Hence, out of the twenty-four piglets, ten were considered to have a good response while fourteen were considered bad responders.

### DNA extraction

DNA was extracted from the nasal and rectal swabs following ZymoBIOMICS protocol, with the following modifications. Microbial cell lysis was performed by adding 700 µL of lysis buffer to 350 µL of sample, and DNA was eluted in 50 µL of elution buffer. DNA concentration was measured with a BioDrop DUO (BioDrop Ltd) and stored at − 80ºC. A negative sample consisting of DNA/RNA shield alone was included as control.

### Detection of* G. parasuis* by PCR

The presence of virulent and non-virulent strains of *G. parasuis* in the nasal samples was confirmed by PCR of the specific *vtaA* leader sequence, as described in Galofré-Milà, et al.^29^, that allows the differentiation of virulent and non-virulent *G. parasuis*.

### Microbiota sequencing

The library preparation and Illumina sequencing were performed at Servei de Genòmica, Universitat Autònoma de Barcelona. Variable regions 3 and 4 (V3-V4) from 16S rRNA gene were sequenced from genomic libraries prepared using Illumina recommended primers for these variable regions of the gene (fwd 5ʹTCGTCGGCAGCGTCAGATGTGTATAAGAGACAGCCTACGGGNGGCWGCAG, rev 5ʹGTCTCGTGGGCTCGGAGATGTGTATAAGAGACAGGACTACHVGGGTATCTAATCC) and following Illumina protocol (Illumina pair-end 2X250 bp, MS-102-2003 MiSeq Re285 agent Kit v2, 500 cycle). Sequenced amplicons lengths were checked on a Bioanalyzer DNA 1000 chip (Agilent).

### Microbiota bioinformatic analysis

The analysis of the 16S rRNA gene amplicons was performed using Quantitative Insights into Microbial Ecology (QIIME) 2 software in its 2023.9 version^30^. After importing the paired-end raw reads, primers were removed with *q2 cutadapt*^31^, with the option to discard any sequence not containing the primers. Reads were quality filtered, denoised, paired-end merged, sorted into Amplicon Sequence Variants (ASVs) and chimera cleaned with DADA2^32^ used as a qiime2 plugin. Additionally, low quality positions at the 3ʹ end of the reads were removed. Three extra filtering steps were applied after DADA2. The first one, to remove non-prokaryotic sequences with *q2 quality control*^33^ by discarding all ASVs that did not match the Greengenes 13_8 database^34^ clustered at 88% identity, after aligning with VSEARCH^35^ under permissive parameters (65% identity and 50% query coverage). Taxonomy was assigned to the remaining ASVs using a scikit-learn naïve Bayes classifier^36^, previously trained against V3-V4 16S rRNA gene region to increase its accuracy^37^ using the same Greengenes 13_8 database (clustered at 99% identity). A second filter was applied to discard all sequences classified as *Archaea*, *Chloroplast* or *Mitochondria*. Thirdly, all ASVs present in the negative control sample were removed from the analysis (21 ASVs; as previously done^27^) using ID-based filtering. The phylogenetic tree was built using FastTree^38^ after aligning the remaining ASVs with MAFFT^39^. Functional genes present in the predicted metagenome were inferred with PICRUST2^40^ and mapped to KEGG database^41^ modules.

The diversity analysis was done at a common depth of 76,253, corresponding to the lowest sampling depth using *q2 diversity*. The alpha diversity of the samples was measured with Shannon^42^ and Chao1^43^ indexes and the beta diversity with Jaccard^44^ and Bray–Curtis^45^ dissimilarity indexes (qualitative and quantitative, respectively). We tested linear correlations between the microbiota diversity and the antibody levels using Spearman correlation coefficient^46^. The correlation of the vaccine response (antibody delta) with alpha diversity indexes was calculated with *cor.test* function in Stats R package^47^ version 4.3.1. Correlation between beta diversity and vaccine response was computed with Mantel test (999 permutations) included in *q2 diversity beta-correlation*^48^. Correlations between taxa/functional modules and antibody response were inferred using Maaslin2 R package ^49^ filtering taxa with less than 0.01 abundance and the rest of the parameters set to default, using abundances normalized relatively per sample as input (Total Sum Scaling). After Benjamini–Hochberg correction, significance was considered when q < 0.05.

In the discrete comparative analysis (good against bad responders), alpha diversity differences between groups were estimated by Kruskal-Wallis tests (999 random permutations) using *q2 diversity alpha-group significance*^50^. In the beta diversity discrete analysis, the Principal Coordinate Analysis (PCoA) was computed with *q2 diversity core-metrics*^51,52^, which was visualized in R with qiime2R package^53^. The significance of beta diversity comparisons was tested by PERMANOVA pairwise tests (999 permutations), and the percentage of explanation of the studied variables using Adonis function from Vegan R software package^54^, using q2 diversity beta-group-significance^55^. For all mentioned tests, *P* values lower than 0.05 were considered significative.

The healthy swine nasal core microbiota was obtained by filtering all ASVs from the most prevalent genera in healthy pigs as described previously^56^. Briefly, all ASVs not classified within the stated genera were filtered out using *q2 feature-table* filtering options. The prevalence threshold to consider a specific genus as core-microbiota was set to 80%. To find taxa differentially abundant on the remaining nasal core-microbiota between good and bad responders we used Lefse^57^ under its default parameters to perform a linear discriminant analysis (LDA). Taxa showing a LDA score > 2 between the two groups were considered as significantly different.

R script language (version 4.2.2) was used in RStudio environment (version 2022.07.0)^58^ to process Qiime2 microbiome generated data as well as to generate plots using qiime2r, ggplot2^59^, tidyverse^60^ and reshape2^61^ packages.

### Statistical modelling

Prior to undertaking statistical analysis, microbiota composition at order level was screened for unlikely or missing values. No data were excluded on this basis. Subsequently, a descriptive statistical analysis was carried out to the nasal and rectal microbiota composition based on the relative abundance of ASVs for both DNA and RNA. We ran two different statistical models of nasal microbiota including a multivariable logistic regression model with the variation (ΔAb) in vaccine response into good (better response) or bad (worse response) responders as the outcome variable and a multivariable linear regression with the actual value of the ΔAb in vaccine response as the continuous outcome variable. This has been applied for both microbiota composition based on nasal and rectal samples. Initially, a univariable model was carried out to test the unconditional associations between dependent and various independent variables (relative abundances of members of the nasal microbiota). Only independent variables with *P* ≤ 0.25 in this initial screening were included in multivariable logistic and linear regression models in accordance with Dohoo et al*.*^62^. To account for the farm variations, an additional model was developed accounting for farm as a random effect for each of nasal and rectal DNA using generalized mixed models. For each model, the significant independent variables from the univariable analysis were then offered to a multivariable model and a manual backward elimination was implemented, to obtain a final model that exclusively included variables with a *P* value < 0.05, considered as significant. The *P* value and the regression coefficient (b) with a 95% confidence interval (95% CI) were reported for each variable. In a similar approach, we developed a model following the same analysis for relative abundances of members of the rectal microbiota. In all statistical analyses, the results were regarded as significant at *P* ≤ 0.05. All statistical analyses were conducted using the R version 3.3.3 software.

## Results

### The increase in antibody levels correlated with increase in nasal microbiota diversity

To examine the response to vaccine administration or to natural exposure to *G. parasuis*, we determined the antibody levels from the piglets before and after vaccination or in equivalent times for those non-vaccinated but naturally exposed to the pathobiont, which was detected by PCR in the nasal samples (Table 1). All piglets showed maternal derived antibodies, although at different levels (farm 2 showed higher maternal antibodies due to sow vaccination). Since the levels of antibodies were diverse at both tested timepoints, the dynamics of the antibody levels (response) were measured using the difference between both values in each animal (delta antibody value, ΔAb). This value was used to preliminary classify the piglets into good or bad responders, i.e., piglets showing variations above the median of the group or below the median, respectively (see “Methods”, Supplementary Fig. 1 and Table 1).

**Table 1:** Level of antibodies against *G. parasuis* (shown as A_450nm_ from ELISA) and presence of virulent (Vir) and non-virulent (Nvir) *G. parasuis* by PCR.

Farm	Vaccination	Antibodies at 1–3 weeks of age	Antibodies at 8–9 weeks of age	Vir PCR	Nvir PCR	Variation (ΔAb)*	Response**
Farm 1	Weeks 3 and 6	0.265	0.65	−	+	0.385	Bad
		0.699	0.682	−	+	− 0.017	Bad
		0.62	1.55	−	+	0.93	Good
		0.837	1.619	−	+	0.782	Good
		0.859	0.933	+	+	0.074	Bad
		0.54	1.496	−	+	0.956	Good
		1.511	0.959	−	+	− 0.552	Bad
		0.757	1.547	−	+	0.79	Good
Farm 2	Weeks 2 and 5	2.083	0.906	−	+	− 1.177	Bad
		2.474	0.719	−	+	− 1.755	Bad
		2.243	0.835	−	−	− 1.408	Bad
		1.927	1.913	+	+	− 0.014	Good
		1.197	0.605	−	+	− 0.592	Bad
		0.656	1.715	−	+	1.059	Good
		1.061	1.138	−	+	0.077	Good
		2.196	1.069	−	+	− 1.127	Bad
Farm 3	Unvaccinated	1.56	0.275	−	+	− 1.285	Bad
		0.448	0.623	+	+	0.175	Good
		0.429	0.322	+	+	− 0.107	Bad
		1.006	0.433	+	+	− 0.573	Bad
		0.363	0.514	+	+	0.151	Good
		0.325	0.682	+	+	0.357	Good
		N.A	0.42	+	+	N.A	Bad
		0.461	0.121	−	+	− 0.34	Bad

*The variation between the level of antibodies at the two timepoints (ΔAb).

**A preliminary classification into good or bad antibody responders (see “Methods”).

*N.A.* not available.

**Fig. 1:**
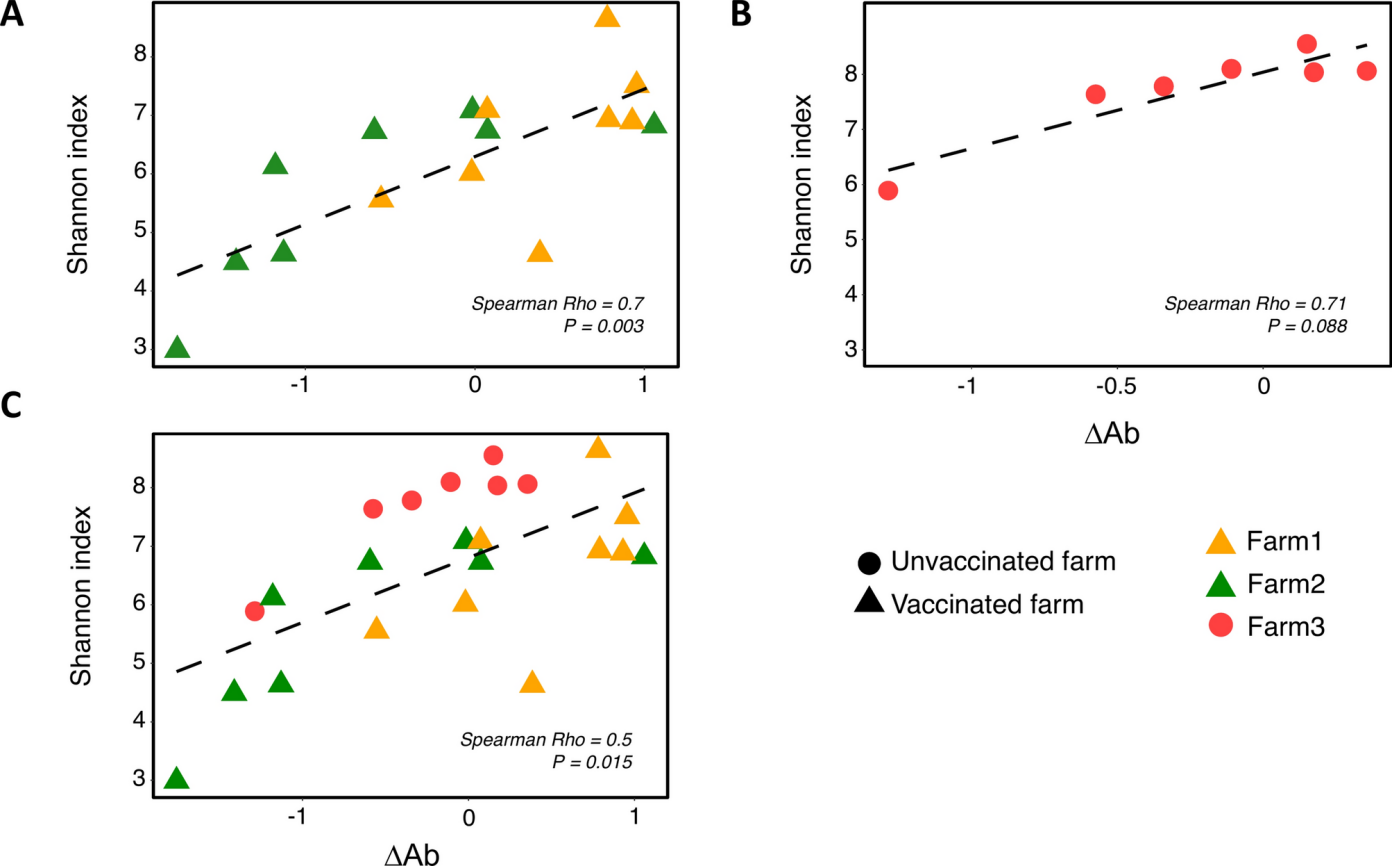
Correlation between nasal microbiota alpha diversity and antibody response. Spearman correlation between alpha diversity of the nasal microbiota (measured by Shannon index) and the antibody response (ΔAb, measured as the difference between the level of antibodies after and before vaccination or equivalent times in non-vaccinated piglets) are shown (**A**) in both farms vaccinated against *G. parasuis* (yellow and green triangles), (**B**) in the unvaccinated farm (red spheres) and (**C**) in the three farms together. Each tendency line depicted in the graphs (dashed lines) was generated using geom_smooth function (ggplot2) using linear model (lm) as the method.

The association between the nasal microbiota composition and the response to vaccination was initially evaluated in samples from the two vaccinated farms. A strong positive correlation of the alpha diversity estimated through Shannon index was observed with the antibody variation levels, ΔAb (Spearman Rho = 0.7, *P* = 0.003, Fig. 1A). A similar but more moderate tendency was observed for Chao1 index (Spearman Rho = 0.48, *P* = 0.06), indicating that piglets with a more diverse nasal microbiota at vaccination time, responded better to vaccination. Regarding the beta diversity analysis, weak and moderate correlations were detected between the qualitative and quantitative distance matrices and the ΔAb (Jaccard and Bray–Curtis Spearman Rho = 0.29 and 0.42, respectively; Mantel test *P* < 0.007), showing that larger community differences between samples (primarily quantitative) were associated with greater differences in the ΔAb.

To examine if the influence of the nasal microbiota was specific for vaccinated animals, we included in the analysis unvaccinated animals with natural exposure to *G. parasuis* (farm 3), since strains of this bacterium, including virulent ones, can colonize the nasal cavity of healthy piglets without causing clinical disease. In fact, virulent and non- virulent strains of *G. parasuis* were detected in piglets from farm 3 by specific PCR. Although we could not detect a significant correlation between the ΔAb and the alpha diversity (Shannon index), probably due to the low number of samples, the tendency observed was similar to that detected in vaccinated piglets, i.e. animals with more diverse nasal microbiota showed increased levels of specific antibodies against *G. parasuis* (Spearman Rho = 0.71, *P* = 0.088, Fig. 1B). When the three farms were analyzed together, the Shannon index correlated positively with the ΔAb (Spearman Rho = 0.5, *P* = 0.015, Fig. 1C), while the Chao1 index showed a weaker tendency (Spearman Rho = 0.38, *P* = 0.07). These findings support that animals with more diverse nasal microbiotas tend to exhibit higher level of antibodies against *G. parasuis* even when they are not vaccinated and naturally encounter the pathobiont. There was a weak correlation between the beta diversity distance matrix and the antibody variation under both assessed metrics, possibly because of the introduction of another farm and therefore, more variation (Jaccard and Bray–Curtis Spearman Rho = 0.27 and 0.28, respectively; Mantel test *P* < 0.004).

### Nasal microbiota taxa associated with response to* G. parasuis*

**Fig. 2:**
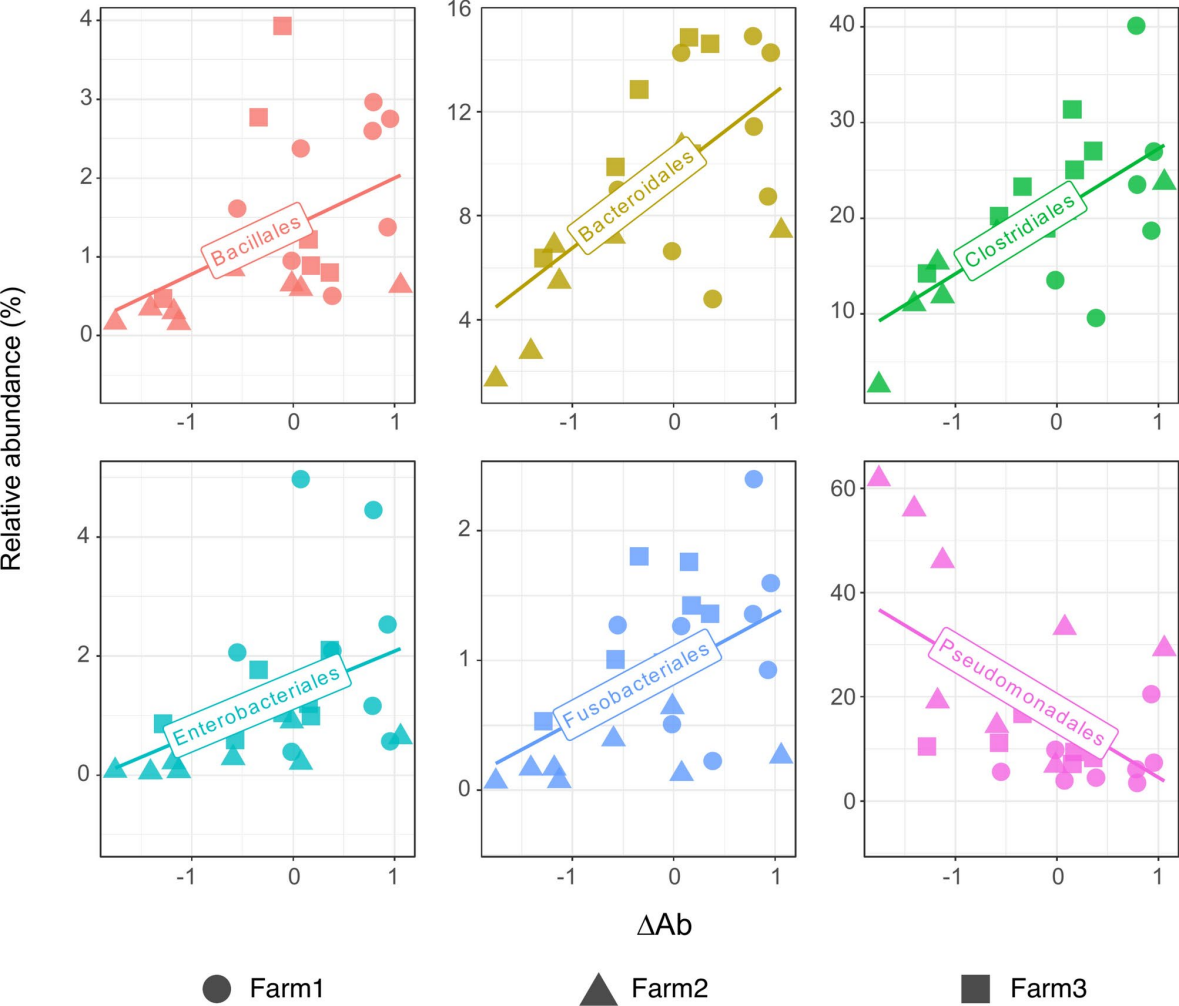
Correlations with the antibody response and nasal microbiota at order level. Scatter plots show the relative abundance versus ΔAb, measured as the difference between the level of antibodies after and before vaccination or equivalent times in non-vaccinated piglets. Only orders found significant with Maaslin2 are shown. Each tendency line depicted in the graphs was generated using geom_smooth function (ggplot2) using linear model (lm) as the method.

To identify nasal microbiota members associated with the antibody response, we investigated the correlations between the taxa and the ΔAb in the three farms. First, we examined if the response to *G. parasuis* correlated with the abundance of this bacterium in the nasal microbiota of the piglets and found no association (Supplementary Fig. 2). When we analyzed the global microbiota composition, six orders and 22 genera showed to correlate with the ΔAb, most of them positively. *Clostridiales* and *Bacteroidales* were the most abundant orders identified with positive correlation with the ΔAb (q < 0.05, Fig. 2). Other orders were also positively correlated, namely *Enterobacteriales*, *Bacillales* and *Fusobacteriales* (q < 0.05, Fig. 2 and Supplementary Table 1), while the only order that correlated negatively with the ΔAb was *Pseudomonadales* (q = 0.043). Correlations at genus level also followed the same dynamics observed for the corresponding orders (Supplementary Fig. 3 and Supplementary Table 1), such as *Prevotella* (*Bacteroidales*) and several members of *Clostridiaceae*, *Lachnospiraaceae*, *Ruminococcaceae* and *Veillonellaceae* families (belonging to order *Clostridiales*), which correlated positively with the delta antibody. Other genera with a positive correlation with the antibody variation were *Klebsiella*, *Staphylococcus, Fusobacterium*, *Pasteurellaceae* (uncl.), *Escherichia*, *Moraxella,* and *Corynebacterium*. Within *Pseudomonadales*, only a divergent *Moraxella* (originally classified as *Enhydrobacter* in the used database but confirmed as *Moraxella* by BLASTn) negatively correlated with ΔAb (see Supplementary Table 1 for all correlations). To further investigate whether these taxa associations with the antibody response might also be reflected in the microbiota functionality, the association between the inferred metagenome functional modules and the ΔAb was also evaluated. Three modules were found to be positively associated with ΔAb (q < 0.05), i.e., iron complex transport system, glycolysis, and simple sugar transport system (Supplementary Table 1).

Some of the previous associations at order level were also identified using multivariable linear regression with the actual value of the antibody variation (ΔAb) as a continuous outcome variable, including two models with and without farm as a random effect which results are shown on Table 2. Again, the presence of *Bacteroidales*, *Clostridiales,* together with *Spirochaetales* in the nasal mucosa of piglets was positively associated with the antibody response to *G. parasuis*. Whereas the presence of *Pseudomonadales* in the nasal mucosa of piglets was negatively associated with the antibody response to *G. parasuis*.

**Table 2:** Nasal microbiota members associated with the variation in antibody response (measured as a continuous outcome variable) to *G. parasuis* (*P* ≤ 0.05) in 23 animals in three farms using a mixed effects generalized linear regression model.

Variable	Coefficient estimate	95% CI	*P* value*
*Pseudomonadales*	− 1.77	(− 4.02)–0.48	0.016
*Bacteroidales*	11.71	(− 12.99)–36.41	0.005
*Enterobacteriales*	18.27	(− 26.36)–62.91	0.845
*Clostridiales*	5.03	(− 6.52)–16.57	0.028
*Erysipelotrichales*	− 216.01	(− 468.99)–36.98	0.518
*Fusobacteriales*	89.13	(− 31.95)–210.21	0.272
*Spirochaetales*	161.34	(− 79.71)–402.39	0.037
*Desulfovibrionales*	− 229.34	(− 591.80)–133.13	0.172
*Coriobacteriales*	− 116.41	(− 427.39)–194.59	0.099
*Rhizobiales*	− 195.29	(− 491.74)–101.16	0.087

*Taxa with *P* > 0.05 were kept in the final model due to significant confounding effect with other significantly associated taxa.

### Rectal microbiota diversity also correlated with antibody response

Different microbiotas can crosstalk with the immune system and have a systemic effect. Since previous studies have highlighted the role of the gut microbiota with the antibody response, we aimed to analyze whether similar associations to those detected between the nasal microbiota and the antibody response were also occurring in the rectal microbiota. The alpha diversity of the rectal microbiota of animals from the three farms moderately correlated with the antibody response (Spearman Rho = 0.44 and 0.6; *P* = 0.036 and 0.003 for Chao1 and Shannon indexes, respectively, Supplementary Fig. 4). The beta diversity of the rectal microbiota was weakly correlated with the ΔAb (Jaccard and Bray–Curtis Spearman Rho = 0.21; Mantel test *P* < 0.006). No significant correlations were found between taxa in the rectal samples and the ΔAb using Maaslin2, except for a *Mogibacteriaceae* unclassified genus (*Clostridiales*), positively associated with the ΔAb (q = 0.021). Nevertheless, four orders were identified as significantly correlating with the delta antibody in both multivariable linear regression models using the ΔAb as a continuous outcome variable (accounting for farm variations as a random effect or not). The presence of *GMD14H09* and *Clostridiales* in the rectal microbiota of piglets was associated with better antibody response to *G. parasuis,* whereas the presence of *Pasteurellales* and *Enterobacteriales* was negatively associated with the antibody response to *G. parasuis* (Supplementary Fig. 5 and Table 3). The farm effect was negligible.

**Table 3:** Rectal microbiota members associated with the variation in antibody response (as a continuous outcome variable) to *G. parasuis* (*P* ≤ 0.05) in 23 animals in three farms using a mixed effects generalized linear regression model.

Variable	Estimate	95% CI	*P* value
*Clostridiales*	0.301	(− 2.327)–2.929	0.0075
GMD14H09	76.900	2.731–151.069	0.036
*Pasteurellales*	− 42.895	(− 84.076)–(− 1.715)	0.036
*Enterobacteriales*	− 4.045	(− 7.247)–(− 0.842)	0.016

### Microbiota associations with piglets classified as good antibody responders

To further investigate and validate the associations with a good antibody response, we used the preliminary classification according to the ΔAb (see “Methods”, Supplementary Fig. 1 and Table 1).

**Fig. 3:**
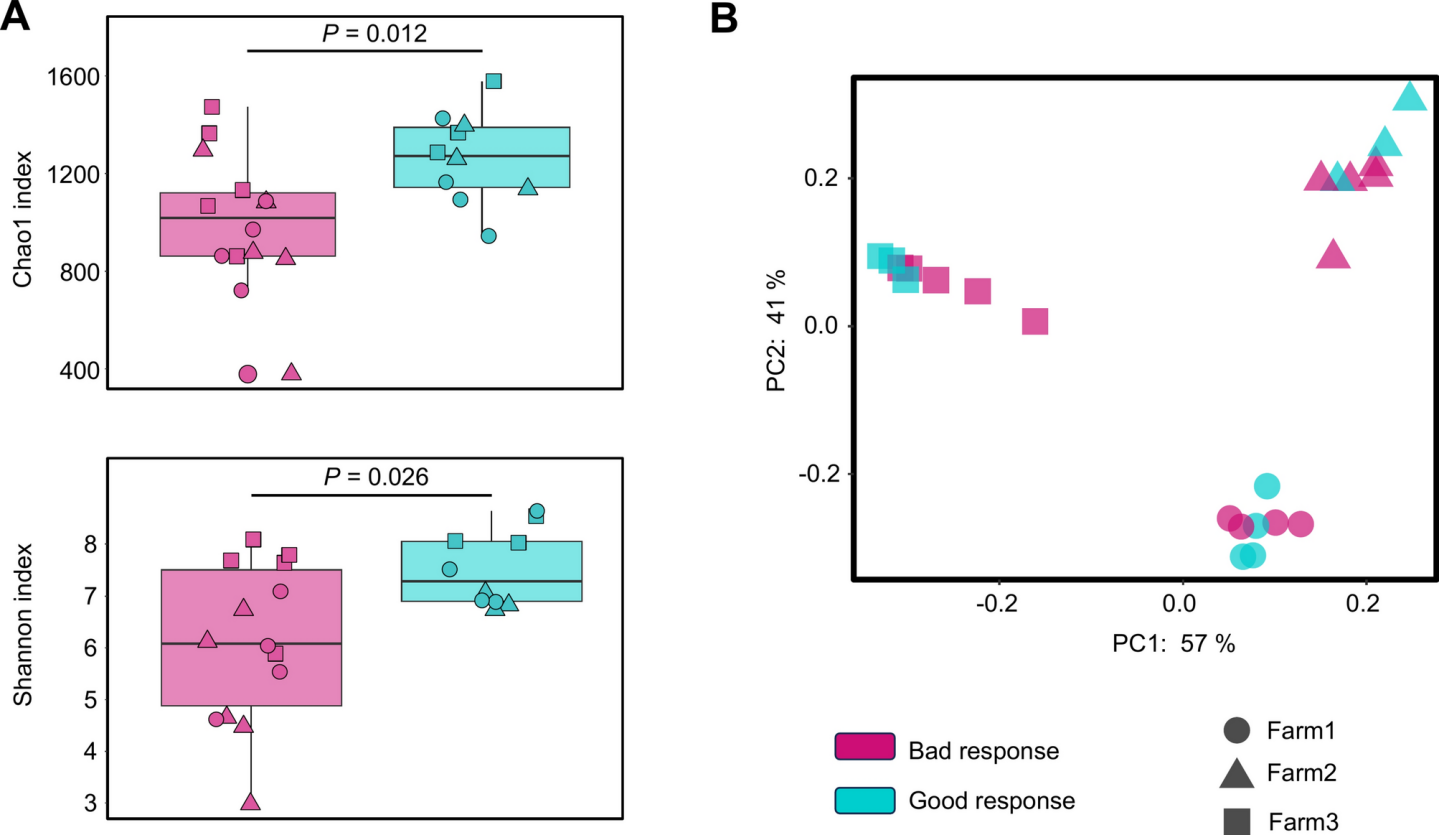
Alpha and beta diversity of nasal microbiota from good (turquoise) and bad (mauve) responders. (**A**) Alpha diversity measured by Chao1 and Shannon indexes. (**B**) Beta diversity estimated through Bray–Curtis dissimilarity index. The shapes indicate different farms.

Using this classification, a more diverse and richer microbiota was observed in the good responders compared to the bad ones (Shannon and Chao1 indexes, *P* < 0.05; Fig. 3A). In the beta diversity analysis, no significant differences were detected between good and bad responders. A strong environmental effect caused by the analysis of three different farms together was observed in the clustering of the samples, quantified to be 29% qualitatively and 48% quantitatively (Adonis test R^2^ using Jaccard and Bray–Curtis indexes, respectively, *P* = 0.001, Fig. 3B). However, the differences between responders were still non-significant when this effect was evaluated as a nested variable in the PERMANOVA, considering farm as the main effect.

**Fig. 4:**
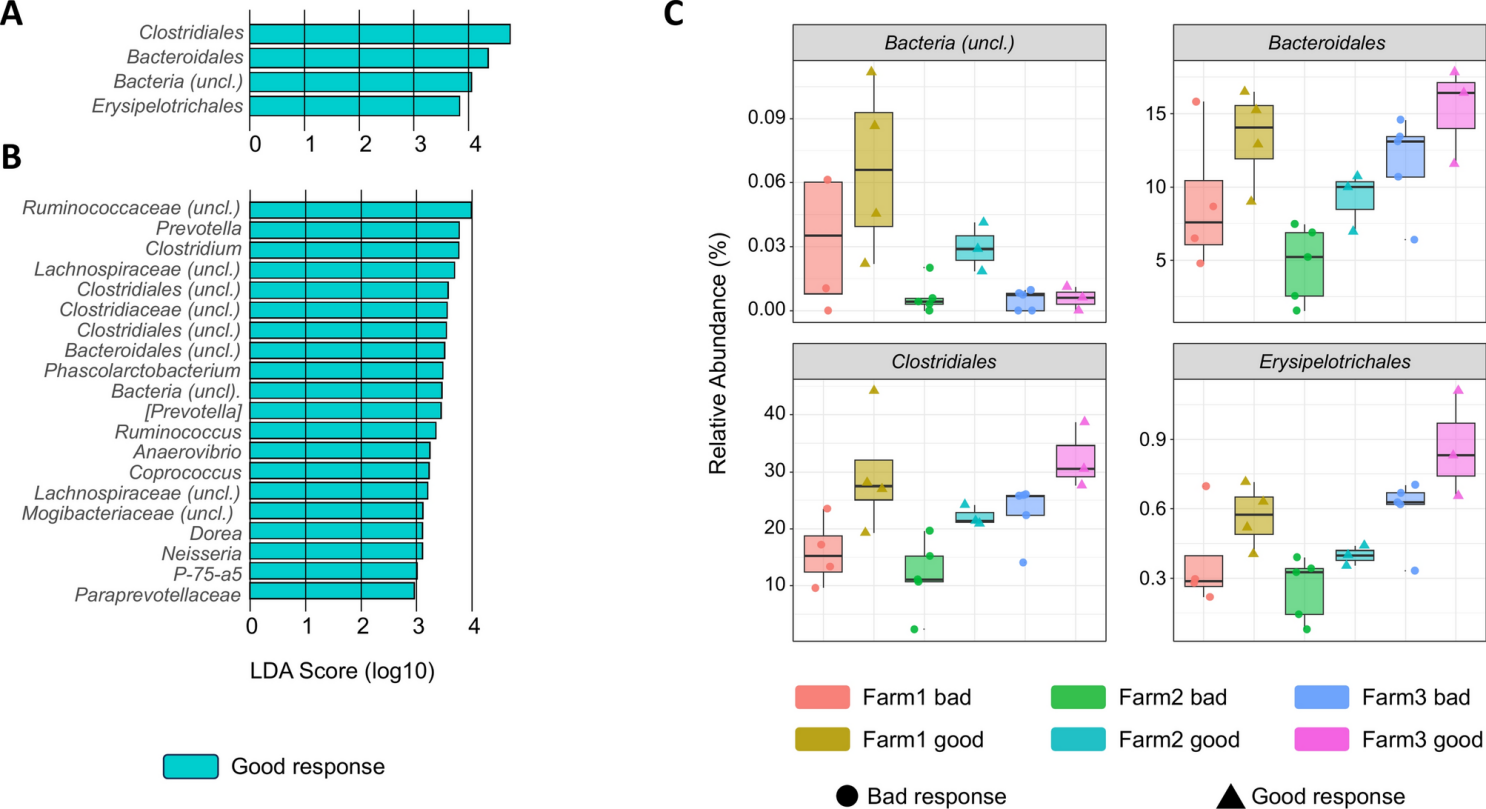
Nasal microbiota taxa discriminating good responders. (**A**) At order level. (**B**) At genus level. (**C**) Relative abundance of the orders identified as associated with good responders (**A**) in the nasal microbiota. The analysis was done using Lefse. No taxa discriminating bad responders were found.

With the aim to reduce the effect of the variability among farms and knowing that is frequent to detect transient environmental taxa in nasal microbiota samples, the ASVs from genera not included in the core of the swine nasal microbiota were filtered out (see “Methods”). Interestingly, the significance of the alpha diversity comparison between good and bad responders increased (Shannon and Chao1 *P* < 0.009) indicating that differences observed were not caused by transient taxa and truly relied on common swine nasal colonizers. To reveal nasal taxa that were associated with good or bad responders to *G. parasuis* beyond the farm environmental effect*,* the filtered microbiota composition from all samples was submitted to linear discriminant analysis (Lefse) at different taxonomic levels (Fig. 4 and Supplementary Fig. 6). In nasal samples, four orders and 20 genera from the core of the nasal microbiota were found to significantly discriminate good responders (LDA score > 2), independently of the farm of origin. Among the detected taxa, *Clostridiales* and *Bacteroidales* were also found to be associated with good response in the previous analysis using the numeric antibody delta, while *Erysipelotrichales* was only detected in this discrete analysis (Lefse). Most genera associated with good vaccination response belonged to the above orders, with several genera of the *Ruminococcaceae* and *Lachnospiraceae* families (among other *Clostridiales*), as well as *Prevotella* (*Bacteroidales*). *Neisseria* was also associated with the group with better response. No taxa with a LDA score > 2 were associated with a bad response.

Regarding the rectal microbiota, no differences were detected in alpha, beta diversity or taxonomical composition when good and bad responders were compared.

## Discussion

In this study, we evaluated the swine nasal and rectal microbiota composition according to the response to vaccination against *G. parasuis* or natural exposure to this bacterium. Despite all animals in this study being healthy and, therefore, no major changes were expected in their microbiota composition, several differences were identified according to the antibody response. To our knowledge, this is the first study that correlates the porcine nasal microbiota composition as a predictor for the response to vaccination providing some notable findings that highlight the relationship between the diversity of the microbiota and the immune response. Moreover, the introduction of an unvaccinated control farm emphasized that these claims could also stand for animals that naturally encounter pathogens.

The initial levels of antibodies were variable between farms, but also within the same farm indicating variable level of maternal immunity at the initial sampling time of the piglets (1–3 weeks of age). Nevertheless, independently of the starting level of antibodies in each farm, piglets exhibited variable dynamics in the levels of antibodies. To analyze the antibody response, and also deal with variability between animals, we decided to measure the response to *G. parasuis* using the difference between the two time points i.e., after and before vaccination or in equivalent times for the unvaccinated farm (delta antibody value, or ΔAb).

In agreement with previous reports^8,13^, piglets in this study with more diverse microbiota showed higher antibody response. In the case of nasal samples, these differences were clearer when transient taxa were removed from the analysis (by keeping the healthy core microbiota), highlighting that the major drivers were swine nasal commensals. Moreover, the fact that stronger correlations were observed when alpha diversity was measured with Shannon index rather than Chao1 suggests that not only species richness but also community evenness are important for better immune responses. We found three metabolic modules inferred from the microbiota composition that were positively correlated with the antibody response, that can possibly be related with an increased alpha diversity (these three modules may be associated with more bacterial activity). Several investigations targeting the swine microbiota in different scenarios also reported higher alpha diversity in healthy groups compared to different diseased animals or environments^63–65^ and associated it with a better immune status of the animals. The well-known notion that the microbiota participates in the maturation of the immune system^7^ also agrees with these results. Contrarily, other studies on gut microbiota did not report any relationship between alpha diversity and response to vaccination^11,16,18,19,24,25^. This apparent contradiction may be explained by different factors, such as the time when the microbiota was evaluated, the type of vaccine or immune stimulus or the host studied, which all can affect the output of the analyses.

Since samples came from three different environments (farms), we did not focus the analysis on ASVs, but searched for associations at higher taxonomic levels (order and genus). We detected several taxa in the nasal cavities of the piglets that positively correlated with a higher increase in the level of antibodies. Among these, taxa frequently found in the gut microbiota (*Bacteroidales* and *Clostridiales*) appeared as the most associated. It is not unusual to find these taxa in the swine respiratory tract^27,66^, most are included in the nasal core, and their presence has been recently discussed in a previous study from our group that confirms these bacteria are indeed present in this body site^27^. Nevertheless, the role of these gut microbiota-related taxa in the upper respiratory tract is still uncertain^27,67^, and future studies focusing on these microbes may help understanding their connection with the immune response. Interestingly, genera within these two orders appeared in higher abundances in farms without respiratory disease condition compared to farms with Glässer’s disease^63^, as well as when compared with farms with polyserositis caused by *Mycoplasma hyorhinis*^64^. Besides these, other taxa frequently found in the swine nasal microbiota, such as *Fusobacterium*, *Staphylococcus*, *Pasteurellaceae* (uncl.), *Moraxella* and *Neisseria*^66^ were positively associated with the increase of antibodies against *G. parasuis*. The presence of these taxa may be important for the immune system stimulation and therefore, for the immune response. *Moraxella* has already been proved as a potential nasal probiotic within a five-bacteria cocktail^68^. However, very few nasal colonizers are being considered for the moment as probiotics to enhance the immune response^69^, which deserve further investigation. Regarding taxa negatively correlated with the antibody response, only a different group from *Moraxella* genus negatively correlated with *Glaesserella* antibody response. In a previous study^63^, higher abundances of *Moraxellaceae* (*Moraxella* and *Enhydrobacter*) were found in farms with Glässer’s disease compared to control farms. The variability within the genus *Moraxella* may explain the different role in health status depending on the species or even the specific strain, as it has been previously observed regarding the virulence of these bacteria^70^. Similarly, in gut microbiota, immune stimulation by probiotics is highly influenced by variables like the specific strain, host or environment (also explaining differences across farms in this study)^71^.

In the case of rectal samples, fewer taxa were identified as related with the response to vaccination, possibly because this microbiota can be more resilient to exposure to environmental taxa compared to the nasal microbiota. Two gut-microbiota core orders (*Clostridiales* and *Enterobacteriales*)^72^ were identified as positively and negatively correlated with the antibody response, respectively. Munyaka, et al. showed that in vaccination against *Mycoplasma hyopneumoniae*, Operational Taxonomic Units (OTUs) from *Bacteroidales* (mainly *Prevotella*) found in the fecal microbiota at the moment of vaccination were positively correlated with higher antibody titters^24,25^. OTUs classified within *Clostridiales* were associated with both high and low responses, while no associations within *Enterobacteriales* were found. The fact that in these studies samples came from the same environment (farm) and the analyses were performed at OTU level could explain the differences with this study (three farms and associations evaluated at higher taxonomical levels). On the other hand and in agreement with our results, studies on the role of the human microbiota also associated several taxa within *Clostridiales* with better immune responses^8,12,13,18^, possibly through the production of short-chain fatty acids that stimulate the immune system^8,73^. However, other studies found taxa within *Clostridiales* associated with immune responses below average^19^, suggesting differences in the role that taxa within this order may have. Aligned with our findings, higher abundances of *Enterobacteriaceae*^16^ and *Enterobacteriales*^14^ were associated with lower responses to vaccination. In any case, although bacteria within *Clostridiales* are frequently associated with health, while some *Enterobacteriales* can be associated with disease^74^, both orders contain taxa that contribute metabolically to immune system homeostasis and stimulation^11,75^. Two lower abundance orders (GMD14H09 and *Pasteurellales*) were also identified in this study as positively and negatively associated with the antibody response, respectively; but their presence and role are unclear in this microbiota. Finally, despite not finding any associations within *Bacteroidetes* and *Actinobacteria*, such as *Prevotella* and *Bifidobacterium*, these orders may play a role in immune response in humans and pigs^11,13–15,24,25^, and also deserve further study.

There are some limitations to be considered in this study. The main limitation is the variation given by the three farms, which introduced substantial environmental variability complicating the analysis. Similarly, there may be a certain caveat in comparing the antibody responses between animals from different farms (variable antibody levels and dynamics, maternal immunity, etc.). To address these issues, we classified the piglets as good or bad responders within each farm using a fixed criterion. The discrete analysis using this classification into good or bad responders agreed with the results obtained in the numeric correlation analysis between the microbiota composition and the level of antibodies, which otherwise proved to be a fruitful strategy with higher statistical power, as expected. Also, in order to diminish the limitation of not accounting for collinearity in the regression models, the variables were examined for the possibility of interaction and confounding effects. Nevertheless, other statistical models could be explored to improve the analysis of these high-dimensional and compositional data. Finally, we analyzed the microbiota composition before weaning, when it may not be yet stable, giving rise to further variability. However, the fact that we obtained significant results using these samples can also be considered a strength, as the associations found can be considered more representative since they were observed in three different farms with independent management.

In conclusion, this study highlights the importance of the diversity of the nasal and rectal microbiotas in the antibody response when facing an antigen (naturally or in vaccination). The associations of several bacterial species with a better response may serve as a starting point to investigate how these taxa stimulate the immune system from the nasal cavity. Additionally, it may help to design targeted interventions to enhance immunization and protection in animals.

## Supplementary Information

Supplementary Figures.

Supplementary Tables.

## Data Availability

The raw sequencing data used in this study can be found at NCBI’s SRA database under BioProject ID PRJNA981084.
