# Supplementary material for: Pig nasal and rectal microbiotas are involved in the antibody response to Glaesserella parasuis
Source: Sci Rep. 2025 Jan 17;15:2347. doi: 10.1038/s41598-025-85867-6 (PMC11742689; doi:10.1038/s41598-025-85867-6)
Supplement: Supplementary file 1 — Supplementary Figures. [file 41598_2025_85867_MOESM1_ESM.pdf]

Pig nasal and rectal microbiotas are involved in the antibody response to *Glaesserella parasuis*

Pau Obregon-Gutierrez[1,2,3], Yasser Mahmmod[4], Emili Barba-Vidal[5], Marina Sibila[1,2,3], Florencia Correa-Fiz[1,2,3, *], Virginia Aragón[1,2,3,*]

[1] Unitat mixta d'Investigació IRTA-UAB en Sanitat Animal, Centre de Recerca en Sanitat Animal (CReSA), Campus de la Universitat Autònoma de Barcelona (UAB), Bellaterra 08193, Catalonia, Spain

[2] Institut de Recerca i Tecnologia Agroalimentàries, Programa de Sanitat Animal, Centre de Recerca en Sanitat Animal (CReSA), Campus de la Universitat Autònoma de Barcelona (UAB), Bellaterra 08193, Catalonia, Spain

[3] WOAH Collaborating Centre for the Research and Control of Emerging and Re-Emerging Swine Diseases in Europe (IRTA-CReSA), 08193 Bellaterra, Catalonia, Spain

[4] Department of Veterinary Clinical Sciences, College of Veterinary, Medicine, Long Island University, 720 Northern Boulevard, Brookville, NY 11548, USA

[5] HIPRA, Avda. la Selva, 135, 17170 Amer (Girona), España

*Co-corresponding authors

Supplementary Figure 1

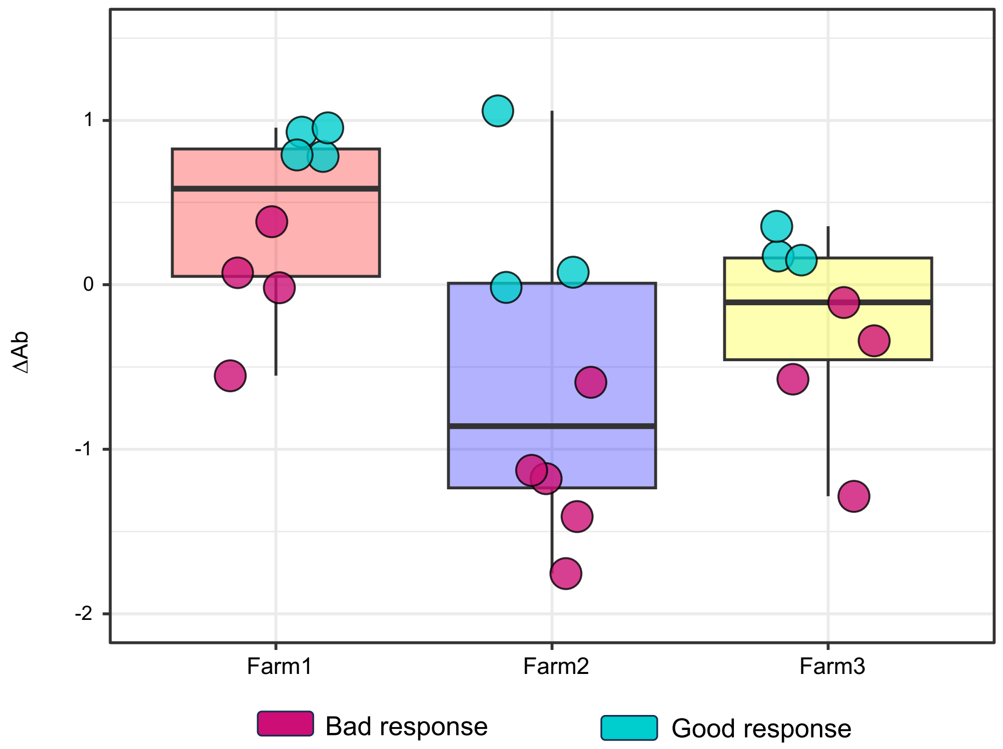

Supplementary Figure 1: Antibody variation of the samples under study (ΔAb). The antibody variation is measured as the difference between the level of antibodies after and before vaccination or equivalent times in non-vaccinated piglets) shown per farm. Classification into good (turquoise) or bad (mauve) responders is also provided.

Supplementary Figure 2

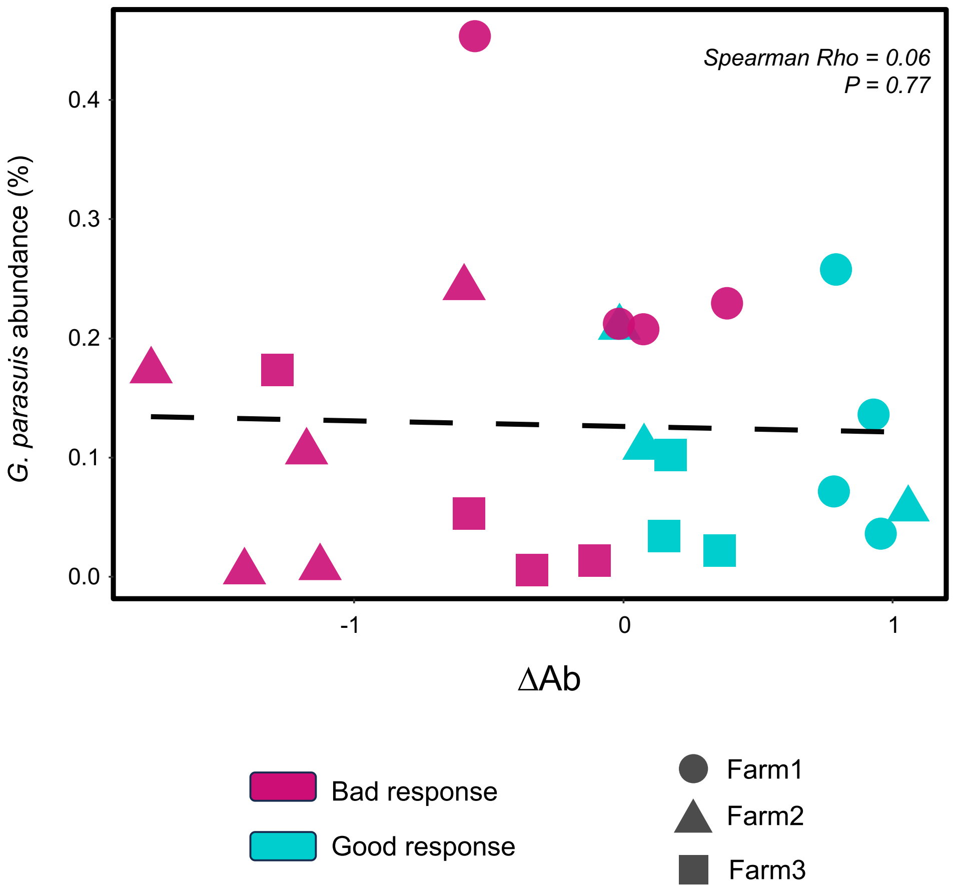

Supplementary Figure 2: Correlation between *G. parasuis* and antibody response. Spearman correlation between *G. parasuis* relative abundance (all ASVs collapsed) and antibody response (ΔAb measured as the difference between the level of antibodies after and before vaccination or equivalent times in non-vaccinated piglets) are shown for nasal microbiota samples from the three farms. The tendency line depicted in the graph (dashed line) was generated using geom_smooth function (ggplot2) using linear model (lm) as the method.

Supplementary Figure 3

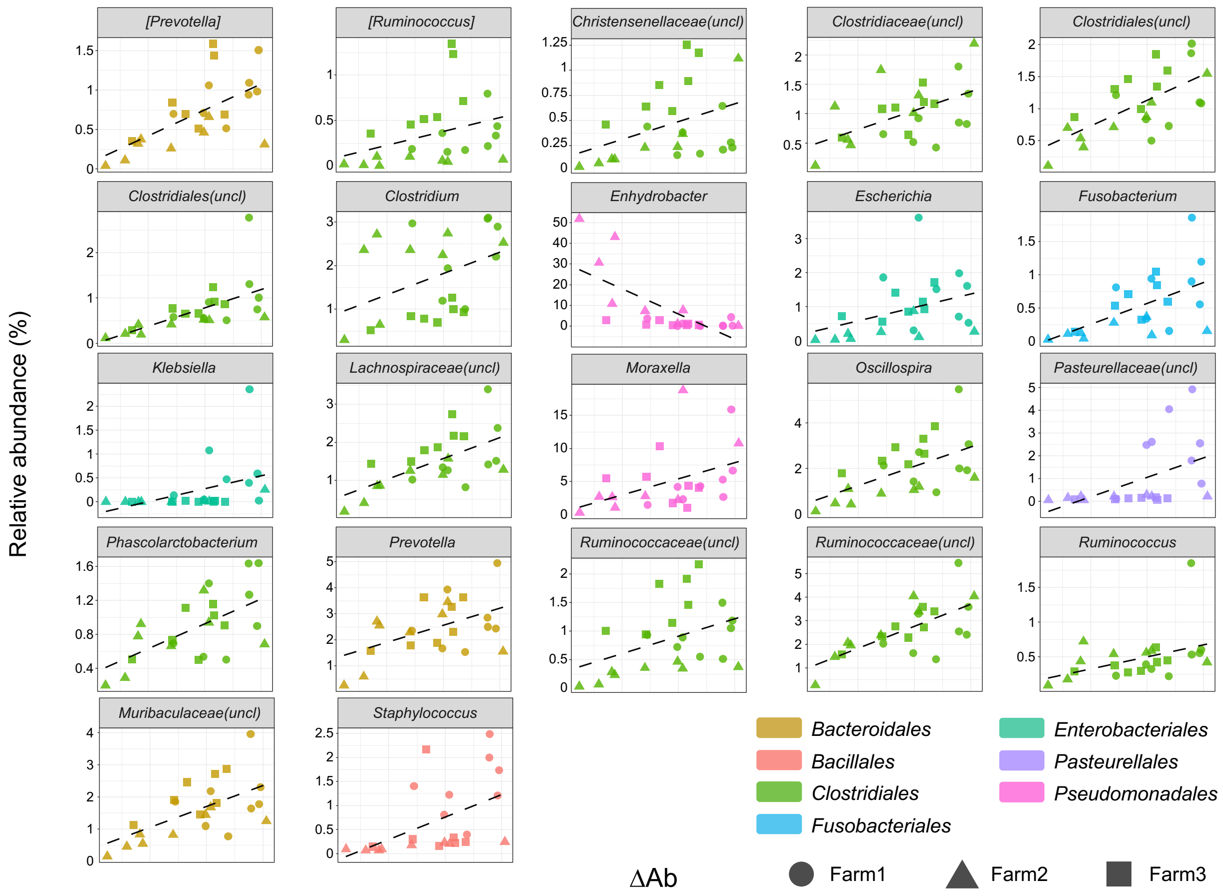

Supplementary Figure 3: Correlations with the antibody response and nasal microbiota at genus level. Scatter plots show the relative abundance versus ΔAb, measured as the difference between the level of antibodies after and before vaccination or equivalent times in non-vaccinated piglets. Only genera found significant with Maaslin2 are shown. Each tendency line depicted in the graphs (dashed lines) was generated using geom_smooth function (ggplot2) using linear model (lm) as the method. Color code corresponds to orders englobing these genera.

Supplementary Figure 4

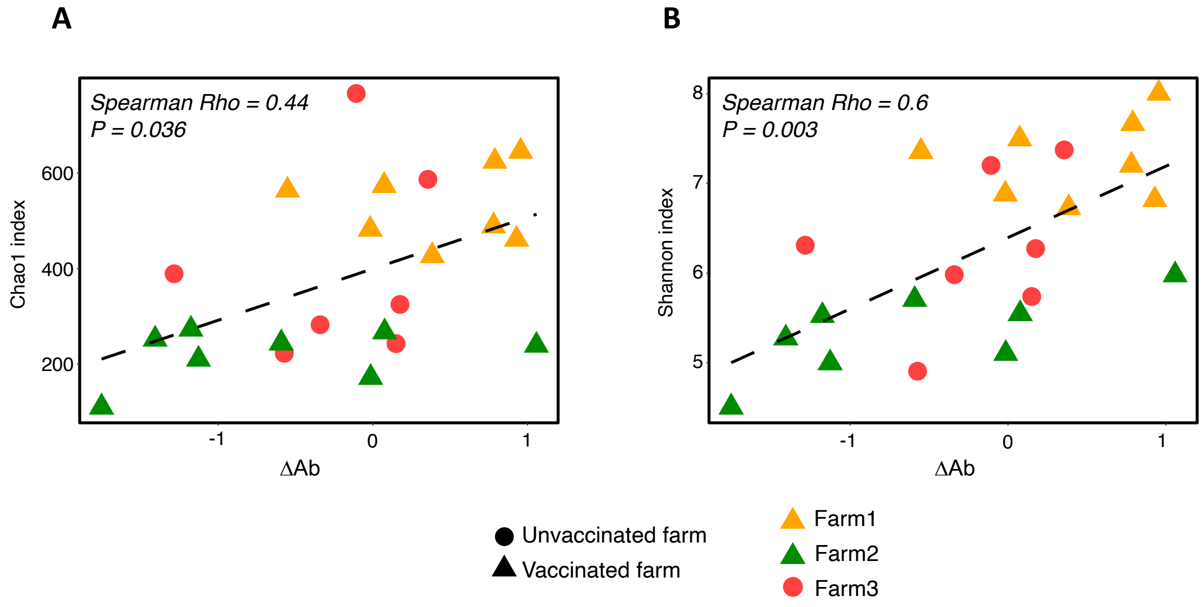

Supplementary Figure 4: Correlation between rectal microbiota alpha diversity and antibody response. Spearman correlation and the tendency as a linear model (dashed lines) between alpha diversity in the rectal microbiota samples are shown, measured by Chao1 (A) or Shannon (B) indexes, and the antibody response (measured as the difference between the level of antibodies after and before vaccination or equivalent times in non-vaccinated piglets, or ΔAb). Each tendency line depicted in the graphs (dashed lines) was generated using geom_smooth function (ggplot2) using linear model (lm) as the method.

Supplementary Figure 5

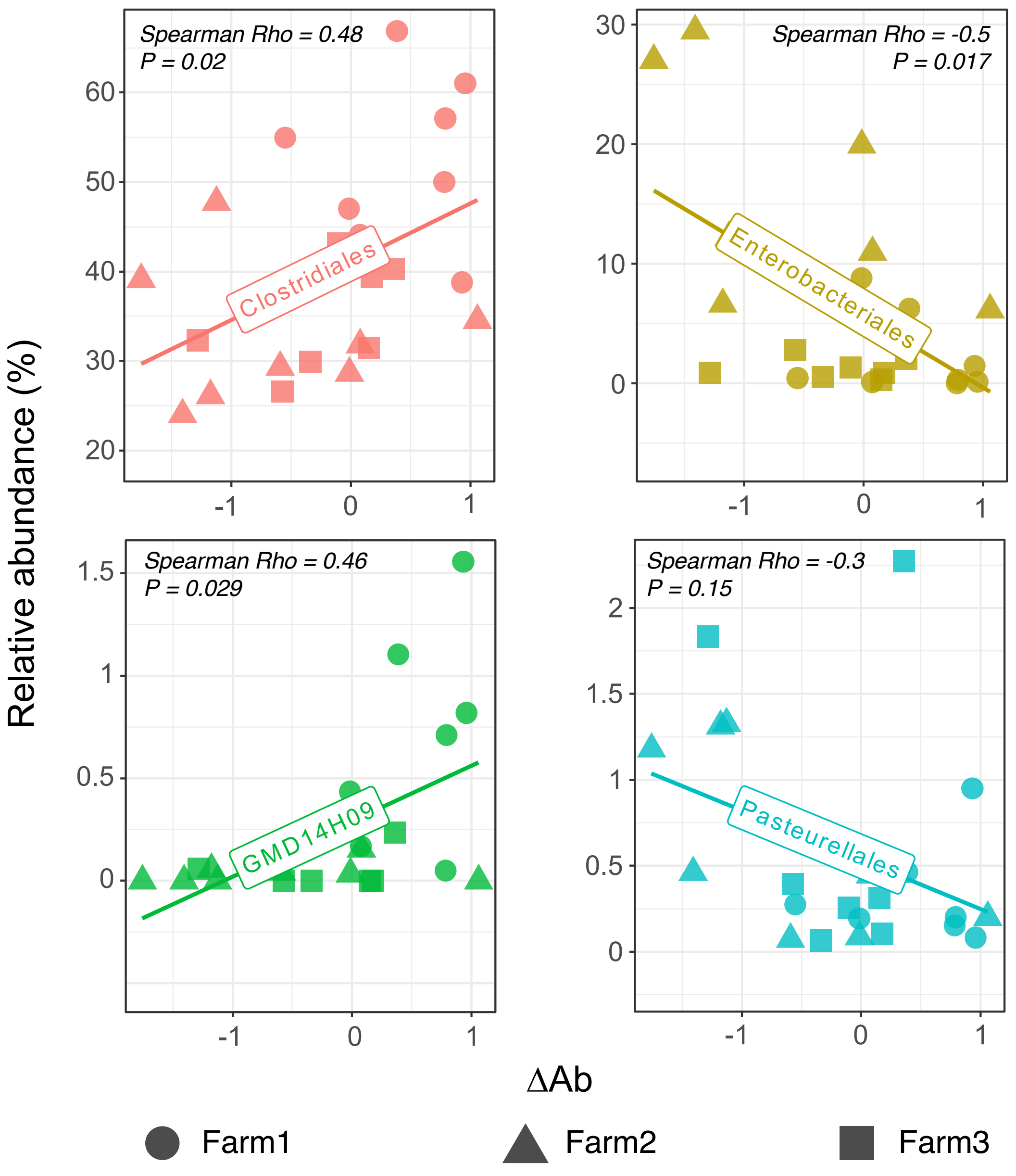

Supplementary Figure 5: Correlations with the antibody response and rectal microbiota at order level. Scatter plots show the relative abundance versus ΔAb, measured as the difference between the level of antibodies after and before vaccination or equivalent times in non-vaccinated piglets. Only orders found significant with Maaslin2 are shown. Each tendency line depicted in the graphs was generated using geom_smooth function (ggplot2) using linear model (lm) as the method. Spearman correlations values are also depicted in each plot.

Supplementary Figure 6

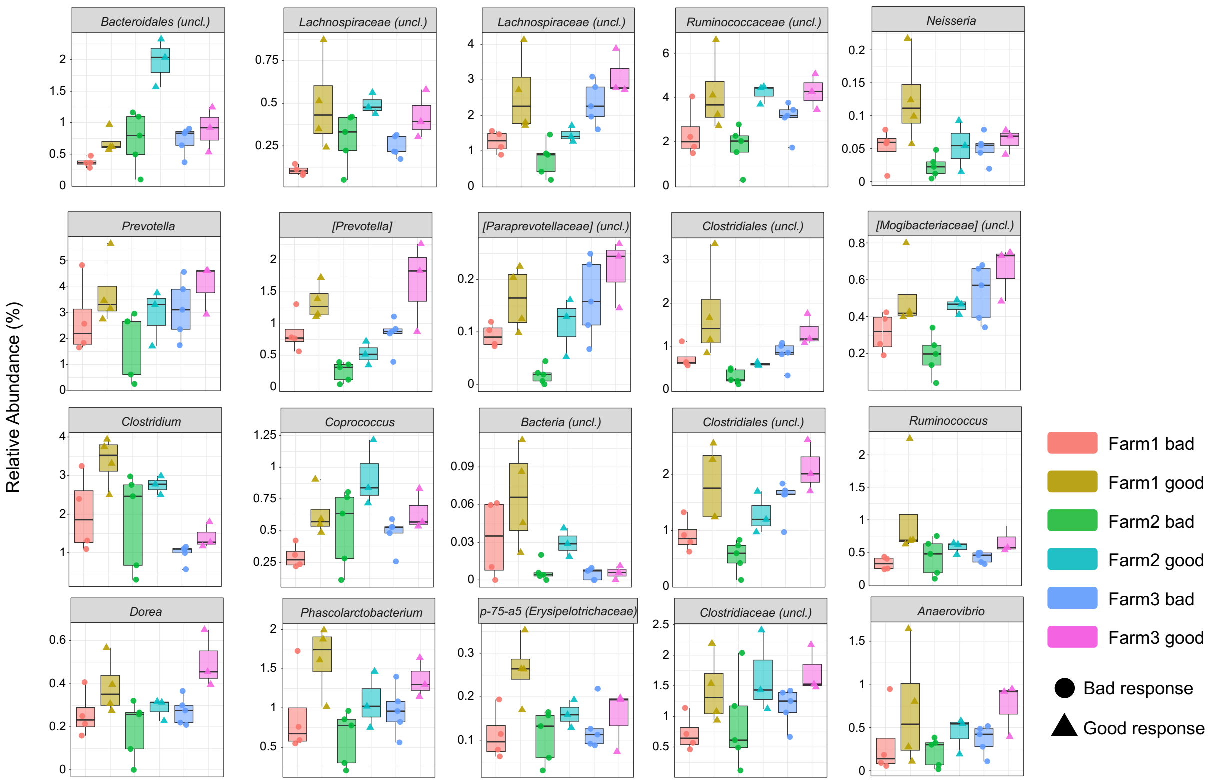

Supplementary Figure 6: Nasal microbiota taxa discriminating good responders at genus level. Relative abundance of the genera identified as associated with good responders in the nasal microbiota in the discrete analysis using Lefse (Figure 4B) are shown.
